# Point of care detection of SARS-CoV-2 antibodies and neutralisation capacity—lateral flow immunoassay evaluation compared to commercial assay to inform potential role in therapeutic and surveillance practices

**DOI:** 10.3389/fpubh.2023.1245464

**Published:** 2023-09-28

**Authors:** Jonathan McGrath, Laura O'Doherty, Niall Conlon, Jean Dunne, Gareth Brady, Aya Ibrahim, William McCormack, Cathal Walsh, Lisa Domegan, Shane Walsh, Claire Kenny, Niamh Allen, Catherine Fleming, Colm Bergin

**Affiliations:** ^1^Department of Genitourinary Medicine and Infectious Diseases (GUIDe), St. James's Hospital, Dublin, Ireland; ^2^Department of Immunology, St. James's Hospital, Dublin, Ireland; ^3^Department of Clinical Medicine, Trinity College, Dublin, Ireland; ^4^Trinity College, Trinity Health Kidney Centre, Trinity Translational Medicine Institute, St. James's Hospital, Dublin, Ireland; ^5^Department of Clinical Medicine, Trinity Translational Medicine Institute, School of Medicine, Trinity College Dublin, Dublin, Ireland; ^6^Health Protection Surveillance Centre (HPSC), Dublin, Ireland; ^7^St. James's Hospital, Dublin, Ireland; ^8^Department of Infectious Diseases, University Hospital Galway, Galway, Ireland

**Keywords:** SARS-CoV-2, point of care, lateral flow immunoassay, sero-epidemiology, neutralisation, antibody

## Abstract

**Introduction:**

As the COVID-19 pandemic moves towards endemic status, testing strategies are being de-escalated. A rapid and effective point of care test (POCT) assessment of SARS-CoV-2 immune responses can inform clinical decision-making and epidemiological monitoring of the disease. This cross-sectional seroprevalence study of anti-SARS-CoV-2 antibodies in Irish healthcare workers assessed how rapid anti-SARS-CoV-2 antibody testing can be compared to a standard laboratory assay, discusses its effectiveness in neutralisation assessment and its uses into the future of the pandemic.

**Methods:**

A point of care lateral flow immunoassay (LFA) detecting anti-SARS-CoV-2 spike (S)-receptor binding domain (RBD) neutralising antibodies (Healgen SARS-CoV-2 neutralising Antibody Rapid Test Cassette) was compared to the Roche Elecsys/-S anti-SARS-CoV-2 antibody assays and an *in vitro* surrogate neutralisation assay. A correlation between anti-spike (S), anti-nucleocapsid (N) titres, and *in vitro* neutralisation was also assessed.

**Results:**

1,777 serology samples were tested using Roche Elecsys/-S anti-SARS-CoV-2 assays to detect total anti-N/S antibodies. 1,562 samples were tested using the POC LFA (including 50 negative controls), and 90 samples were tested using an *in vitro* ACE2-RBD binding inhibition surrogate neutralisation assay. The POCT demonstrated 97.7% sensitivity, 100% specificity, a positive predictive value (PPV) of 100%, and a negative predictive value (NPV) of 61% in comparison to the commercial assay. Anti-S antibody titres determined by the Roche assay stratified by the POC LFA result groups demonstrated statistically significant differences between the “Positive” and “Negative” LFA groups (*p* < 0.0001) and the “Weak Positive” and “Positive” LFA groups (*p* < 0.0001). No statistically significant difference in ACE2-RBD binding inhibition was demonstrated when stratified by the LFA POC results. A positive, statistically significant correlation was demonstrated between the *in vitro* pseudo-neutralisation assay results and anti-S antibody titres (rho 0.423, *p* < 0.001) and anti-N antibody titres (rho = 0.55, *p* < 0.0001).

**Conclusion:**

High sensitivity, specificity, and PPV were demonstrated for the POC LFA for the detection of anti-S-RBD antibodies in comparison to the commercial assay. The LFA was not a reliable determinant of the neutralisation capacity of identified antibodies. POC LFA are useful tools in sero-epidemiology settings, pandemic preparedness and may act as supportive tools in treatment decisions through the rapid identification of anti-Spike antibodies.

## Introduction

Host cellular and humoral immune responses are key determinants of clinical outcomes from severe acute respiratory syndrome coronavirus-2 (SARS-CoV-2) infection (1), the causative agent of coronavirus disease 2019 (COVID-19). As the COVID-19 pandemic moves from a Public Health Emergency of International Concern (PHEIC) (2) towards endemic status, testing strategies are being de-escalated in many areas (3). As laboratory assessment declines, rapid and effective point of care (POC) assessment of SARS-CoV-2 immune response can inform clinical decision making and broader epidemiological monitoring of disease.

Infection with SARS-CoV-2 results in the host development of anti-spike (S) and anti-nucleocapsid (N) antibodies (4), while vaccination with COVID-19 vaccines results in the production of anti-S antibodies alone (5). The SARS-CoV-2 spike (S) protein mediates viral entry into the host cell *via* the host ACE2 receptor and is a critical target for neutralising antibodies (NAb). NAb play a key role in primary prevention of infection and viral clearance (6). Crucial targets within the S-protein include the receptor binding domain (RBD) and N-terminal domain (NTD) on the S1 subunit (6). Accurate determination of virus neutralisation is challenging owing to the requirement for biosafety level 3 (BSL3) facilities utilising live SARS-CoV-2 viral models, and as a result, surrogate assays are often adopted (6).

Preventative and therapeutic approaches to the management of SARS-CoV-2 have progressed significantly over the course of the pandemic, with COVID-19 vaccination becoming a cornerstone of the global response (7–9). Therapeutic options for active SARS-CoV-2 infection vary internationally, with agents such as nirmatrevir-ritonavir (Paxlovid) (10–12), remdesivir (Veklury) (13–15), and dexamethasone (16, 17) commonly used. A number of other less-frequently used options are also available including molnupiravir (Lageyvrio) (11, 18, 19) and monoclonal antibody therapies, such as sotrovimab (Xevudy) (20).

Absent or insufficient immune response to COVID-19 vaccination and/or SARS-CoV-2 infection has been associated with poor clinical outcomes (21). Risk factors for insufficient immune response include advanced age, sex (22), haematological or solid malignancy (21, 23), autoimmune disorders (24), organ transplantation (25), and iatrogenic immunosuppression (26). Rapid identification of an immune response to vaccination or infection may be useful in the decision to treat with currently available SARS-CoV-2 therapeutics and in identifying those requiring booster COVID-19 vaccine doses or other preventative interventions.

Sero-epidemiological studies are an important tool in tracking both COVID-19 spread and vaccine responses (27–29) as the disease enters an endemic phase, with concurrent reductions in national testing pathways (30). The determination of anti-S antibody status may be challenging outside of research settings due to the lack of assay availability in routine clinical practice, particularly in low-resource settings (31). Point of care tests (POCT) for the rapid detection of SARS-CoV-2 antibodies with high sensitivity and specificity aid in this logistical difficulty. Lateral flow POCTs for the rapid detection of SARS-CoV-2 antigens in acute infection are widely available (32), and adopting this modality for the detection of anti-SARS-CoV-2 antibody presence provides an opportunity for more widespread SARS-CoV-2 epidemiological investigation.

The Prevalence of COVID-19 in Irish Healthcare Workers (PRECISE) study is a multi-phase, cross-sectional seroprevalence study of anti-SARS-CoV-2 antibodies in Irish healthcare workers (HCW) across two hospital sites. The initial phases of the PRECISE study assessed HCW SARS-CoV-2 epidemiology and seroprevalence prior to the availability of COVID-19 vaccines (October 2020) (33) and subsequent serological responses to primary COVID-19 vaccination (April 2021) (29). A November 2021 study phase (PRECISE 4) was undertaken immediately prior to the roll-out of booster COVID-19 vaccination for HCWs, assessing the durability of SARS-CoV-2 antibody responses and the changing SARS-CoV-2 serological status of HCWs in the era of booster vaccinations (28). The use of a lateral flow POCT for identifying the presence of anti-S-RBD antibodies compared to a widely used commercial assay and a surrogate neutralisation assay was investigated in this study. This report demonstrates how rapid anti-SARS-CoV-2 S-RBD antibody testing can be compared to a standard laboratory assay, discusses its effectiveness in neutralisation assessment and its uses into the future of the COVID-19 pandemic.

## Methods

### Study site

The PRECISE study is undertaken across two large academic teaching hospital sites. The current study was undertaken in one of these two study sites. The hospital site is a large tertiary referral centre in the south inner city of Dublin and is the largest university teaching hospital in Ireland, with ~4,700 employees.

### Study design and participant recruitment

This cross-sectional seroprevalence study of anti-SARS-CoV-2 antibodies in Irish HCWs, undertaken from 10th to 23rd November 2021, utilised point of care test (POCT) lateral flow immunoassays (LFA) to detect anti-SARS-CoV-2 spike-RBD neutralising antibodies. The performance of this LFA was compared to that of a widely used commercial SARS-CoV-2 antibody assay and an *in vitro* surrogate neutralisation assay (below).

HCW participants were recruited via internal hospital communications, emails, and text messages. Written consent was obtained, and participants completed a written questionnaire detailing demographic, work-related, and COVID-19 infection/vaccination information. Participants were then invited to undergo phlebotomy providing serology samples for anti-SARS-CoV-2 antibodies prior to receipt of their booster vaccination dose if they were opting to receive one. Study numbers were assigned to each serology sample and processed pseudonymously.

### Laboratory methods

Serology samples were tested for total (IgG, A, and M) anti-SARS-CoV-2 spike (S) antibodies and anti-nucleocapsid (N) antibodies using Roche Elecsys-S/Elecsys Anti-SARS-CoV-2 assays, respectively. Cutoff points were as per manufacturer specifications following an in-hospital verification process (34).

From the total hospital site serology samples, a randomly chosen selection was tested using the SARS-CoV-2 neutralising Antibody Rapid Test Cassette (Healgen Scientific LLC, Houston, TX, USA). This rapid test utilises a combination of spike protein-receptor binding domain (RBD) antigen-coated gold particles for the detection of neutralising antibodies to SARS-CoV-2 in human sera. This rapid test cassette is a lateral flow immunochromatographic assay based on the principle of competitive binding. LFA anti-spike-RBD antibody results were categorised as “Negative,” “Weak Positive,” and “Positive.” Samples were processed in a diagnostic immunology laboratory and read by two scientists experienced in the interpretation of LFAs, Western blots, and immunoblots according to the manufacturer's instructions. Pre-COVID-19 pandemic serum samples were tested using the POCT to serve as control samples.

The presence of neutralising antibodies capable of blocking the interaction between spike protein-RBD and ACE2 was additionally investigated in a subset of samples via an *in vitro* ACE2-RBD binding enzyme-linked immunosorbent assay (ELISA). This surrogate neutralisation assay demonstrates the extent to which biotinylated ACE2 interaction with spike-RBD is inhibited by antibodies in participant sera and has demonstrated a close correlation with spike-based pseudo-virus infection assays for the assessment of ACE2-RBD binding neutralisation. Full details of this assay including comparator results to other assays are published elsewhere (28, 35) and in Supplementary material A. The sample selection process for the pseudo-neutralisation assay is detailed in Supplementary material A. Results from this surrogate neutralisation assay were compared to the results of the LFA POCT and Roche assays.

### Statistical analysis

Sensitivity, specificity, positive predictive value (PPV), and negative predictive value (NPV) of the Healgen Rapid Test Cassette were determined in relation to the Roche Elecsys-S anti-SARS-CoV-2 antibody assay. The Kruskal–Wallis test with Dunn's multiple comparisons test was used to compare the anti-S antibody titres determined by the Roche Elecsys-S assay to the POCT results, stratified by the “Negative,” “Weak Positive,” and “Positive” sub-categories. The Mann–Whitney U test and Kruskal–Wallis test with Dunn's multiple comparisons test was used to compare the POC LFA results with the *in vitro* pseudo-neutralisation assay results. Spearman rank correlation and Kruskal–Wallis test with Dunn's multiple comparisons test were used to assess the correlation between anti-S/anti-N titre results with the pseudo-neutralisation assay results. Analyses were performed using R (version 4.1.3, R Core Team 2021) and Graph Pad Prism 9 software.

### Ethical approval

Ethical approval for this study was granted by the National Research Ethics Committee (NREC), application number 20-NREC-COV-101-AMEND-2.

### Funding

This study was supported financially by the Irish Health Service Executive (HSE) COVID-19 budget. The funders had no role in the design of the study, data analysis, or the decision to publish.

## Results

**Figure 1:**
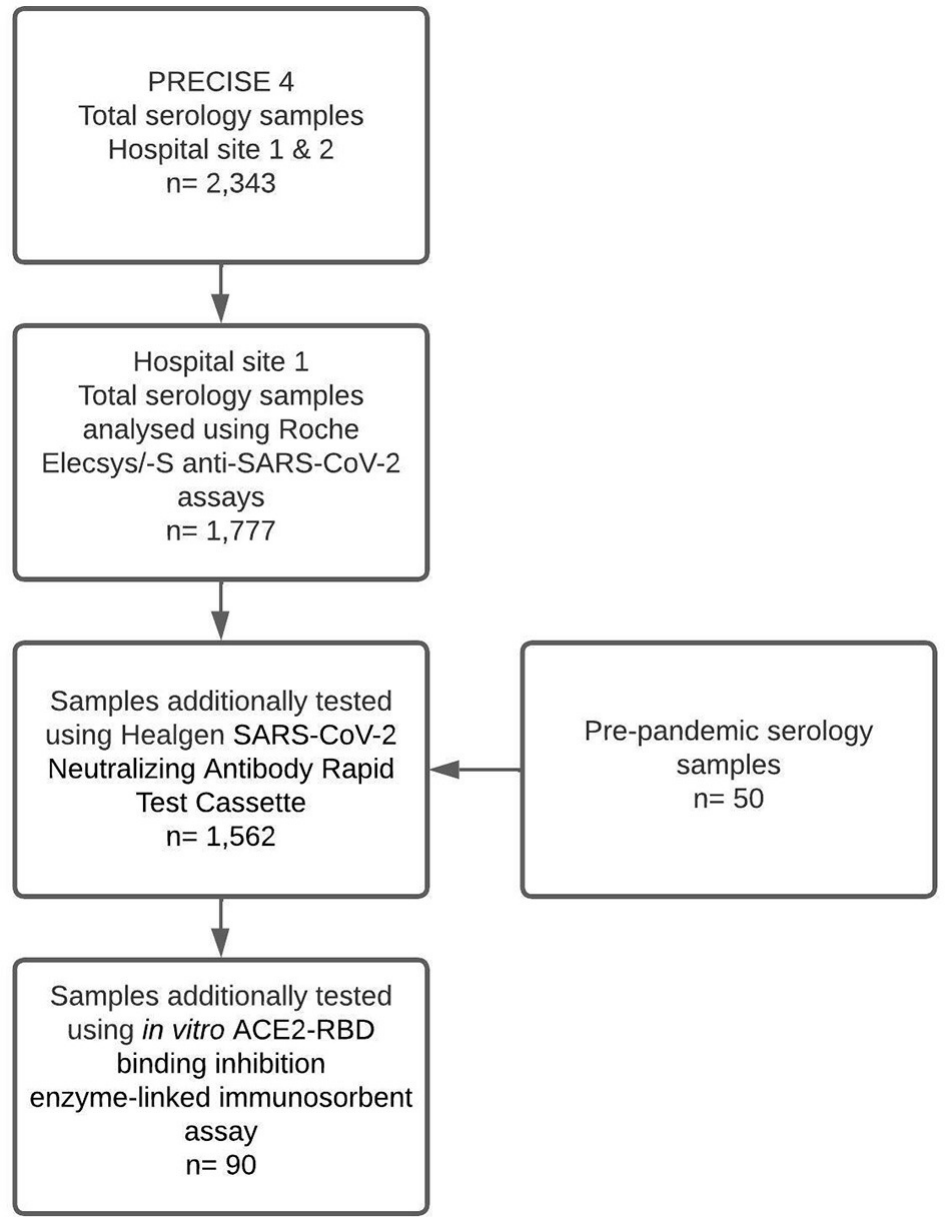
PRECISE 4 sample selection and testing modalities.

Samples from 1,777 participants were tested using the Roche Elecsys/-S anti-SARS-CoV-2 assays to detect total anti-N/S antibodies in the study site. Of those samples, 1,512 were tested using the Healgen SARS-CoV-2 neutralising Antibody Rapid Test Cassette, and 90 were tested using the *in vitro* ACE2-RBD binding inhibition surrogate neutralisation assay. A total of 50 pre-pandemic serology samples were also tested via the Healgen POCT to serve as control samples (Figure 1).

### Demographics

Participant characteristics are presented in Table 1. Of the samples tested using the LFA POCT (*n* = 1,512, excluding control samples), 79% (*n* = 1,199) of participants were female. The median age was 43 years old (IQR 32–51 years). Ninety-nine percent (*n* = 1,497) of participants had received a primary COVID-19 vaccination series with 84.1% (*n* = 1,272) having received the Pfizer/BioNTech COVID-19 vaccine, 14.3% (*n* = 216) receiving the Oxford/AstraZeneca COVID-19 vaccine, 0.6% (*n* = 9) receiving other vaccine brands, and 1.0% (*n* = 15) being unvaccinated.

**Table 1:** Participant characteristics in the lateral flow immunoassay and *in vitro* pseudo-neutralisation sub-cohorts.

**Participant characteristics**		**POC LFA cohort**		**Pseudo-neutralisation cohort**		*P*-value^*^
		**(*****N*** = **1,512)**		**(*****N*** = **90)**		
		***n***	**%**	***n***	**%**	
Age (years)	Mean (SD)	41.9 (11.2)		44.3 (11.4)		**-**
	Median (IQR)	43 (32–51)		45 (34–53)		
Age groups (years)	18–29	266	17.6	13	14.4	0.15
	30–39	354	23.4	19	21.1	
	40–49	457	30.2	22	24.4	
	Over 50	435	28.8	36	40.0	
Sex	Female	1,199	79.3	71	78.9	0.926
	Male	313	20.7	19	21.1	
Ethnicity	Irish (white)	1,162	77.3	69	76.7	0.91
	Any other white background	97	6.5	6	6.7	
	Asian background	233	14.8	13	14.4	
	African and other black background	20	1.3	2	2.2	
Education	Primary	3	0.2	0	0.0	0.101
	Secondary	144	9.5	16	17.8	
	Third level	843	55.8	50	55.6	
	Post-graduate	410	27.1	20	22.2	
	Unknown	112	7.4	4	4.4	
Vaccination status	Vaccinated (primary vaccine series)	1,497	99.0	82	91.1	< 0.001
	Unvaccinated	15	1.0	8	8.9	
Vaccine type	mRNA (Pfizer, Moderna)	1,279	84.6	70	85.4	0.818
	Viral vector (AstraZeneca, Janssen)	217	14.4	11	13.4	
Previous COVID-19	Yes	284	18.8	37	41.1	< 0.001
	No	1,228	81.2	53	58.9	
COVID-19 infection type	Natural infection (pre-vaccine)	196	13.0	30	33.3	0.451
	Post 1st vaccine dose	26	1.7	3	3.3	
	Post 2nd vaccine dose	51	3.4	4	4.4	

Values are *n* and % unless otherwise indicated. POC, point of care; LFA, lateral flow immunoassay; IQR, interquartile range; SD, standard deviation; COVID-19, coronavirus disease-2019. ^*^Calculated using the chi-square test.

### Comparison with the laboratory assay and point of care lateral flow immunoassay

Results are summarised in Table 2. A total of 1,509 (99.8%) samples demonstrated anti-S positivity using the Roche Elecsys-S assay with concordant results in 1,475/1,509 (97.5%) samples analysed *via* the Healgen POCT. Of the positive POCT results, 63/1,475 (4.3%) were deemed “Weak Positive.” All “Weak Positive” POCT results were positive using the Roche assay. Thirty-four samples (2.2%) identified as positive via the laboratory assay returned discordant negative results via POCT. Concordant negative results in both platforms were seen for three samples (0.2%). There were 34 false negative results, and no false positive results on the POCT. All 50 pre-pandemic negative control sera returned negative anti-S results via POCT. The sensitivity of the POCT was 97.7%, and the specificity was 100%. This resulted in a positive predictive value (PPV) of 100% and a negative predictive value (NPV) of 61%.

**Table 2:** Comparison of the Healgen SARS-CoV-2 neutralising Antibody Rapid Test Cassette with the Roche Elecsys-S anti-SARS-CoV-2 assay and pre-pandemic control samples.

	**Healgen point of care antibody test**	**Roche Elecsys-S anti-SARS-CoV-2 spike antibody positive**	**Roche Elecsys-S anti-SARS-CoV-2 spike antibody negative**	**Pre-pandemic control samples**
	***N***	***N***	***N***	***N***
Positive	1,412	1,475	0	0
Weak positive	63			
Negative	87	34	3	50
Total	1,562	1,509	3	50

**Figure 2:**
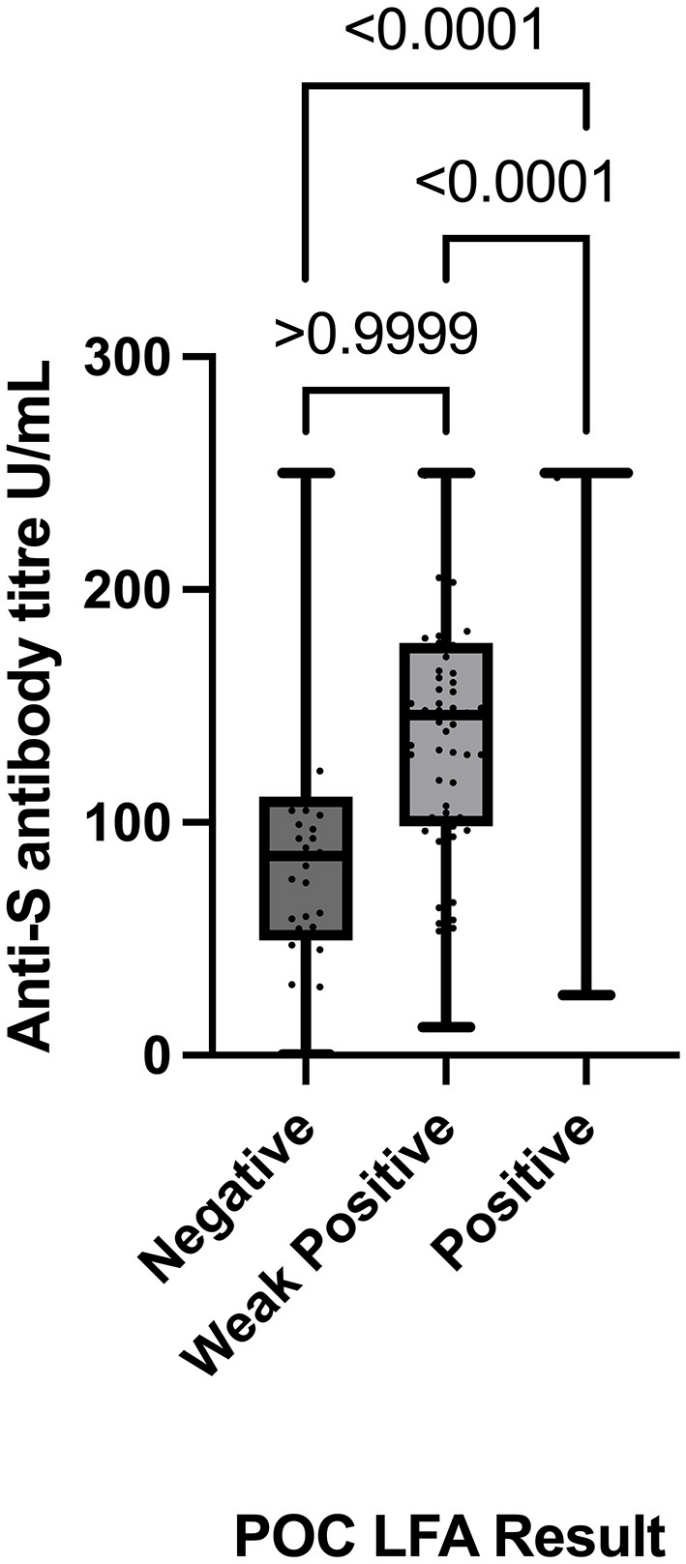
Comparison of POCT results (Negative, Weak Positive, and Positive) with absolute anti-spike titre values (determined by the Roche Elecsys-S anti-SARS-CoV-2 assay) using the Kruskal–Wallis test with Dunn's multiple comparisons test. POC, point of care; LFA, lateral flow immunoassay.

Comparison of anti-S antibody titres determined by the Roche Elecsys-S assay stratified by the POC LFA result groups demonstrated statistically significant differences between the “Positive” and “Negative” LFA groups (*p* < 0.0001) and the “Weak Positive” and “Positive” LFA groups (*p* < 0.0001). There was no statistically significant difference in anti-S titres between the “Negative” and “Weak Positive” LFA result groups (*p* > 0.9999) (Figure 2).

### Comparison of the point of care lateral flow immunoassay with the *in vitro* ACE2-RBD binding inhibition pseudo-neutralisation assay

**Figure 3:**
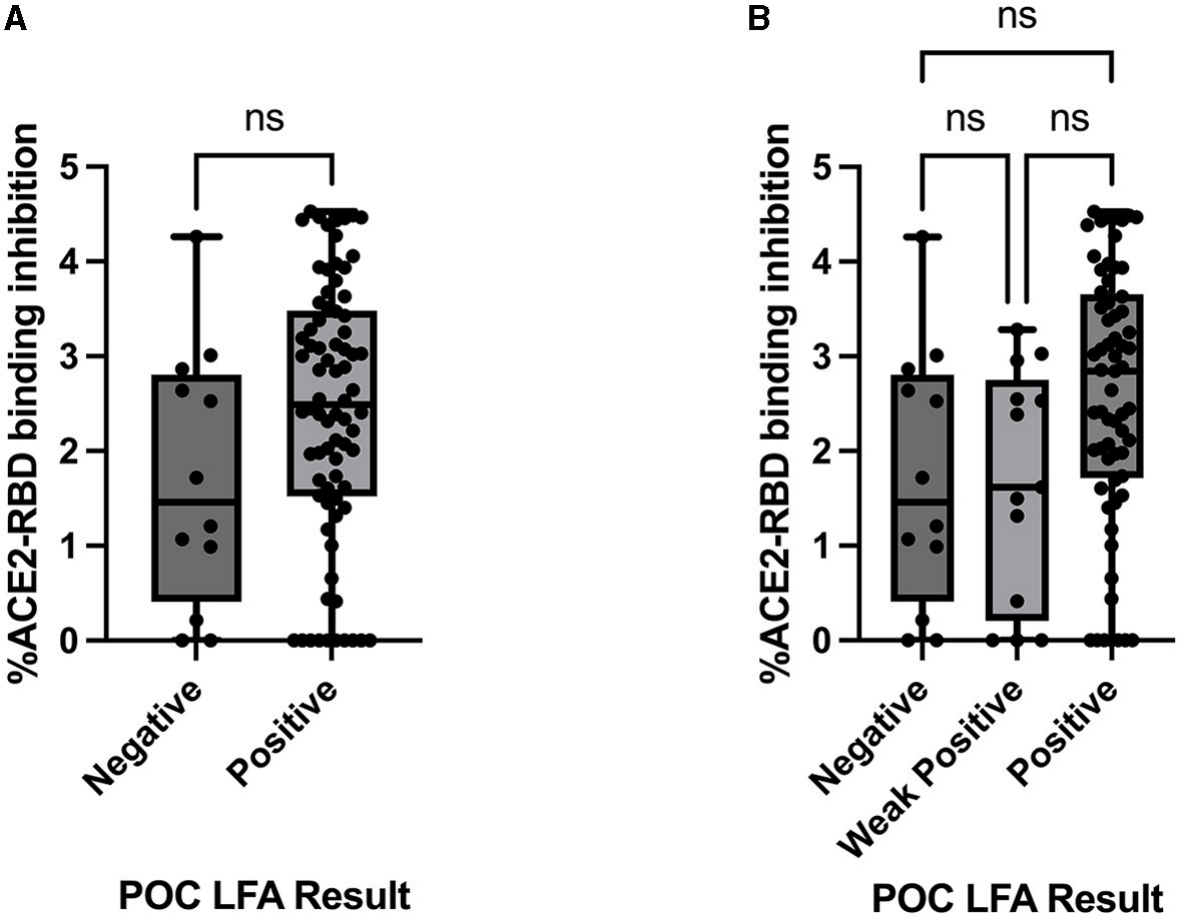
Comparison between the ACE2-RBD binding inhibition assay (percentage inhibition expressed as Log values) and **(A)** Healgen point of care lateral flow immunoassay by result category “Positive” and “Negative,” assessed via the Mann–Whitney U-test and **(B)** Healgen point of care lateral flow immunoassay result sub-categories “Positive,” “Weak Positive,” and “Negative,” assessed via the Kruskal–Wallis test with Dunn's multiple comparisons test. POC, point of care; LFA, lateral flow immunoassay; ns, not statistically significant.

ACE2-RBD binding inhibition via the *in vitro* pseudo-neutralisation assay, expressed as the percentage of ACE2-RBD binding inhibition, was compared to the POC LFA results. There was no statistically significant difference in ACE2-RBD binding inhibition demonstrated when stratified by the LFA POC results either as the total “Positive”/“Negative” result groups or the result sub-categories “Positive,” “Weak Positive,” and “Negative” (Figure 3).

**Figure 4:**
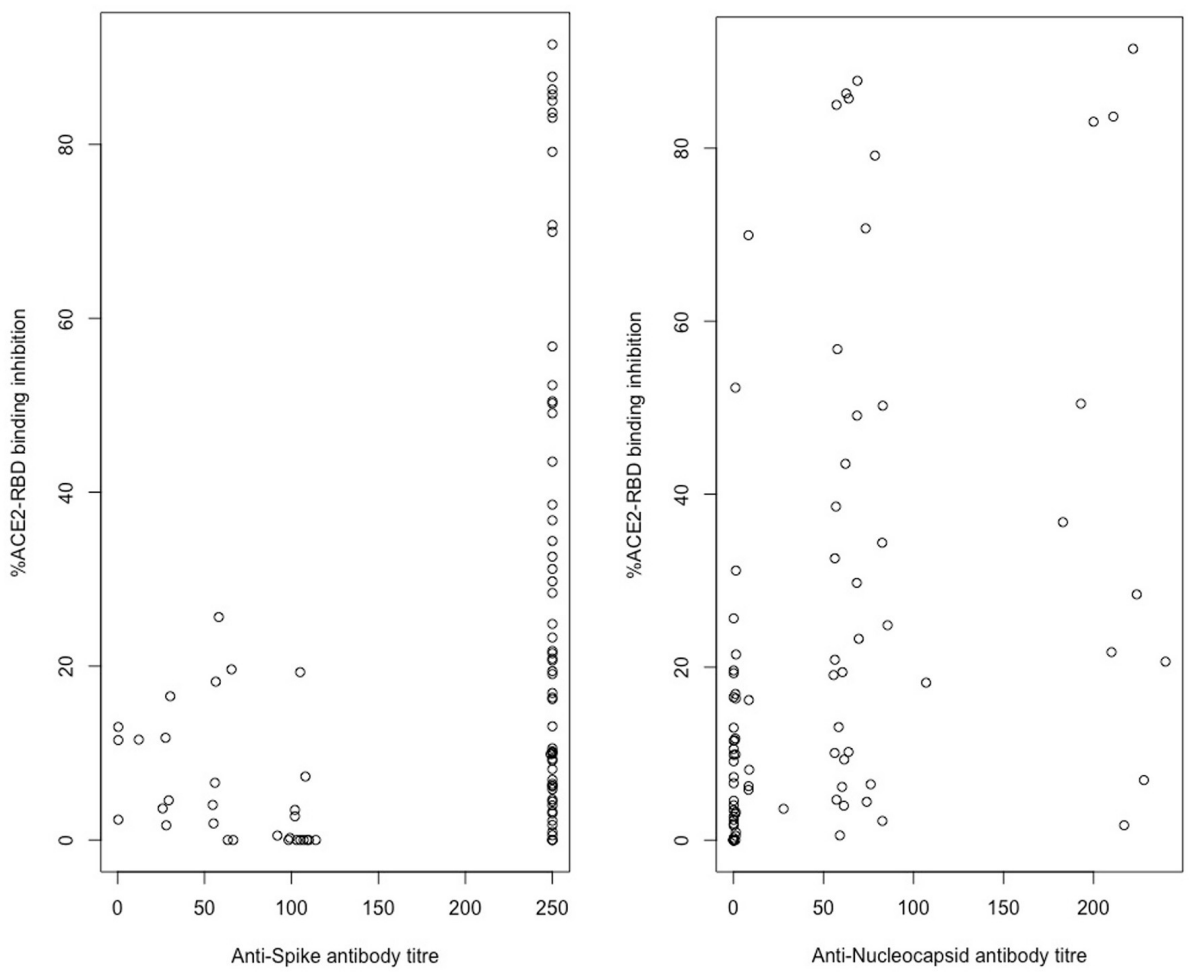
*In vitro* ACE2-RBD binding inhibition correlation with Roche Elecsys/-S anti-spike and anti-nucleocapsid titre results. Assessed via Spearman rank correlation, Anti-S-ACE2RBD (rho = 0.423, *p* < 0.001), Anti-N-ACE2RBD (rho = 0.55, *p* < 0.0001).

**Figure 5:**
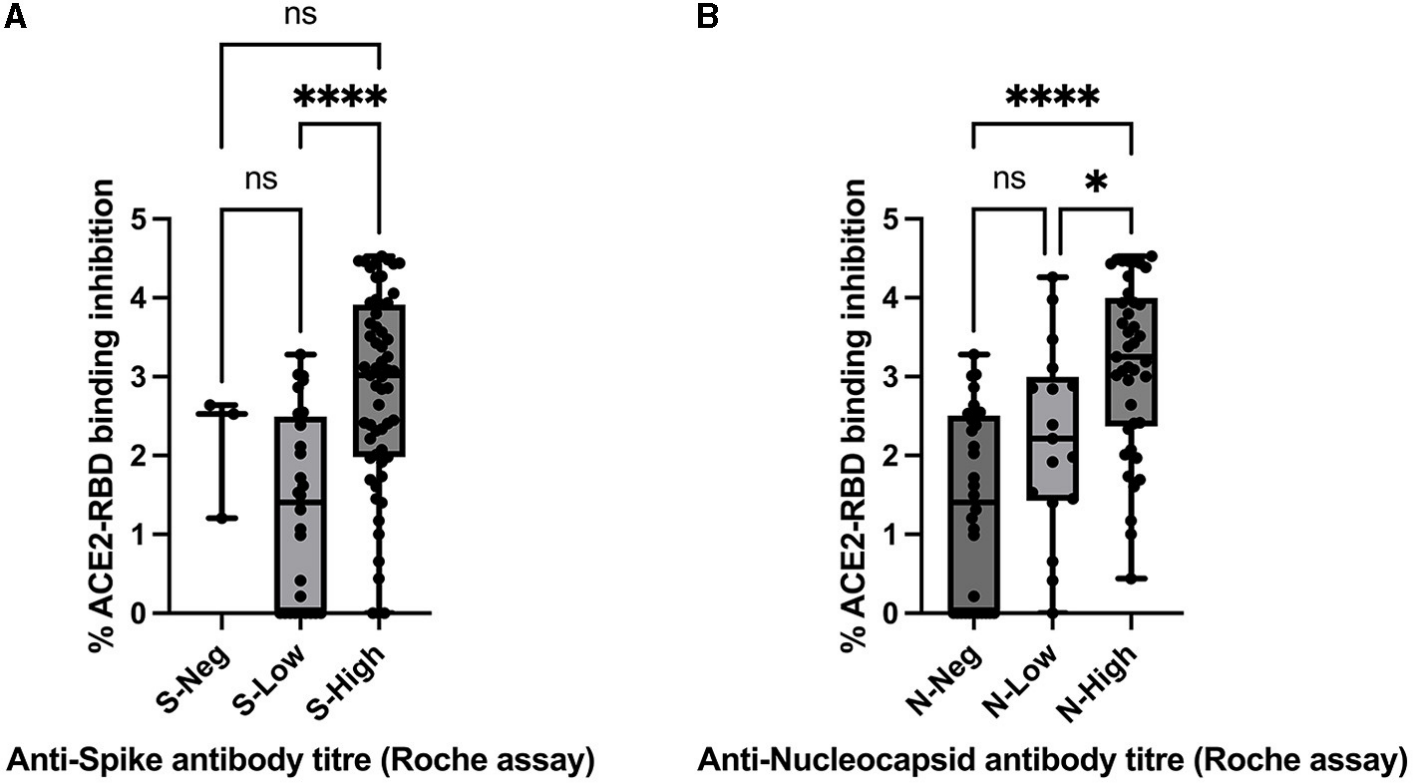
Comparison between ACE2-RBD binding inhibition assay (percentage inhibition expressed as Log values) and **(A)** anti-spike antibody titre via Roche assay by result categories “Negative” (S-Neg), “Low” (S-Low), and “High” (S-High), assessed via the Kruskal–Wallis test with Dunn's multiple comparisons test and **(B)** anti-nucleocapsid antibody titre via Roche assay by result categories “Negative” (N-Neg), “Low” (N-Low), and “High” (N-High), assessed via the Kruskal–Wallis test with Dunn's multiple comparisons test. ^*^ and ^****^ denote statistical significance, ns, not statistically significant.

Spearman's rank-order correlation was used to determine the relationship between the *in vitro* ACE2-RBD binding inhibition pseudo-neutralisation assay results and anti-S antibody titres determined via the Roche Elecsys-S assay with a positive, statistically significant correlation being demonstrated (rho 0.423, *p* < 0.001) (Figure 4). A positive, statistically significant correlation was similarly demonstrated for ACE2-RBD binding inhibition and anti-N antibody titres as determined via the Roche Elecsys assay (rho = 0.55, *p* < 0.0001) (Figure 4). Anti-S antibody titres were additionally sub-classified into “negative” (< 0.8 U/mL, via Roche assay), “low” (>/= 0.8 U/mL– < 250 U/mL), and “high” (>250 U/mL) categories and compared with ACE2-RBD binding inhibition (Figure 5). Statistically significant differences in ACE2-RBD binding inhibition were demonstrated between the “high” vs. “low” sub-categories. Anti-N antibody titres were additionally sub-classified into “negative” (< 1.0 cut-off index, COI, via Roche assay), “low” (>/= 1.0 COI - < 50 COI), and “high” (>50 COI) categories and compared with ACE2-RBD binding inhibition (Figure 5). Statistically significant differences in ACE2-RBD binding inhibition were demonstrated between the “high” vs. “low” and “high” vs. “negative” sub-categories.

## Discussion

We present findings comparing a point of care (POC) lateral flow immunoassay (LFA) for the detection of anti-SARS-CoV-2 spike-RBD antibodies to a widely used commercial assay and an *in vitro* surrogate neutralisation assay. Prior studies have reported favourable comparisons between POCT LFA and immunoassays (36); however, our results compare not only the detection of anti-S antibodies but also assess the relative neutralising capacity as determined by ACE2-RBD binding inhibition on the same samples. The findings demonstrate consistency between the Healgen LFA and Roche Elecsys-S assay, but poor consistency between the LFA and *in vitro* ACE2-RBD protein–protein binding inhibition assay. The findings suggest POC LFAs are a useful tool in the assessment of immune response to infection/vaccination and sero-epidemiological research, but that assessment of neutralisation via this methodology is less effective.

High sensitivity (97.7%) and specificity (100%) were demonstrated in our findings for the detection of anti-S antibodies by the POC LFA when using the commercial Roche assay as a comparative standard. A high PPV and lower NPV were also demonstrated as is expected in a sample population with such high positivity for anti-S antibodies. Pre-pandemic sera were used as negative controls given the high rates of vaccination amongst the HCW staff cohort, and all returned concordant negative results for anti-S antibodies. A large number of samples were tested in comparison to similar investigations, adding to the reliability of our results (37, 38). The sensitivity and specificity of POC LFAs in the detection of anti-SARS-CoV-2 spike antibodies can vary considerably with sensitivity ranging from 53.8% to 98.5% and specificity ranging from 90.9% to 100% (37). Reports have cited sensitivity/specificity of 66.7%−100%/94.2%−99% for the Healgen anti-RBD IgM/IgG Rapid Test Cassette (31, 36, 39). Interpretation of LFA results may be influenced by inter-user variability (31), and our study, similar to some other studies (37), acknowledges this by having more than one independent interpreter of the LFA visual findings. While the aim of LFA is to provide the qualitative detection of a given antibody, we demonstrate statistically significant differences in absolute anti-S titres determined via the Roche assay when stratified by Healgen LFA user-derived result categorisation of “Negative,” “Weak Positive,” and “Positive.” Huang et al. found similar variability with the Healgen Rapid Test Cassette when qualitative LFA anti-spike antibody results were enhanced with the use of a spectrometer to determine additional quantitative data for the detection of NAb (38); the LFA results in our study, however, did not demonstrate a positive correlation with the functional assessment of neutralisation as was demonstrated by Huang et al. (38).

LFA are well suited for use in wider SARS-CoV-2 epidemiological and seroprevalence studies (38). Chemiluminescence immunoassays (CLIAs) such as the Roche Elecys-S assay are not universally available, have cost implications (particularly in low resource settings), require blood draws for serum and transport to CLIA-approved facilities for analysis (36). Point of care LFAs are low cost, require less infrastructure, and use a testing method that the general public has become accustomed to over the course of the pandemic through the diagnosis of acute COVID-19 via POC SARS-CoV-2 antigen testing (32, 40, 41). Consistent findings have been demonstrated when finger-prick blood is used for this modality as an alternative to laboratory-derived serum samples as was used in our study (36). Our findings demonstrate a close correlation between the laboratory assay and the POC test; thus, the Healgen LFA offers a potentially cost-effective way of identifying anti-S positivity while undertaking large-scale SARS-CoV-2 sero-epidemiological studies. Success in the use of POC tests for disease screening, particularly in low-resource healthcare settings, has been demonstrated for many non-coronavirus conditions, such as HIV (42), malaria (43), sickle cell disease (44), and chlamydia (45). This test modality may benefit patients in healthcare settings where laboratory facilities, result turnaround, and follow-up options are limited (46). A test modality that is easy to distribute, perform, and interpret is also beneficial for future pandemic preparedness. As variants of concern continue to emerge (47), the ability to perform large-scale testing in a short period of time is beneficial in identifying outbreaks in at-risk populations.

Absent or insufficient host immune response to COVID-19 vaccination and/or SARS-CoV-2 infection has been associated with poor clinical outcomes (21, 48). Risk factors for insufficient immune response include advanced age, sex (22), haematological or solid malignancy (21, 23), autoimmune disorders (24), organ transplantation (25), and iatrogenic immunosuppression (26) amongst others. Even within these identified at-risk conditions, rates of seroconversion vary widely, with higher overall rates of seroconversion following vaccination in patients with solid tumours (98%) vs. haematologic malignancies (85%) (49). Immune responses following SARS-CoV-2 infection are also reduced in this haematologic group, with multifactorial causes, including receipt of treatment modalities such as anti-CD20 monoclonal antibodies (50). One large cohort study demonstrated seroconversion rates of 69% (51) in haematological malignancy following vaccination although ranges between 16.6% and 84% have been reported (52, 53). The decision to treat a patient diagnosed with acute COVID-19 with anti-viral therapies such as nirmatrevir-ritonavir (Paxlovid) is influenced by the likelihood of that individual having generated immunity following COVID-19 vaccination/SARS-CoV-2 infection (54). The availability of a reliable POC LFA to assess evidence of SARS-CoV-2 antibody presence following vaccination or infection, as demonstrated in our findings, may be used as one component of the clinical decision to treat with such medications, particularly in primary care settings where many of these clinical decisions are now made.

The receptor binding domain (RBD) on the S1 subunit of the spike protein is a known target for neutralising antibodies (NAb), which play a key role in infection prevention and viral clearance due to the role spike-RBD-ACE2 binding plays in SARS-CoV-2 entry into cells (6). The Healgen Rapid Test Cassette LFA detects anti-S-RBD antibodies, with the *in vitro* pseudo-neutralisation assay determining the extent to which ACE2-RBD protein–protein binding is inhibited by antibodies in participant sera. Some reports have suggested that the subclassification of POC LFA results can correlate with functional neutralisation (38); however, this effect was achieved with the aid of a dedicated spectrometer and not user-interpretation, as is the usual use-case with this test modality. In our findings, there was no significant association demonstrated between the degree of ACE2-RBD protein–protein binding inhibition and the POC LFA result when stratified either by “Positive”/“Negative” result categories or by the user-determined “Negative”/“Weak Positive”/“Positive” LFA result sub-categories. This suggests that determining the qualitative presence alone of NAb is not enough to assess functional neutralisation capacity, and other factors, such as antibody titre levels, influence this effect. Indeed, this is further suggested in our findings given that there was a statistical correlation between total anti-S (IgG/A/M) titres determined via the Roche assay and the degree of ACE2-RBD inhibition demonstrated via the *in vitro* assay (assessed via Spearman rank correlation), consistent with other reports demonstrating a correlation between SARS-CoV-2 NAb and “virus-specific” IgG levels (55). A statistically significant difference in ACE2-RBD binding inhibition was also demonstrated when stratified by the anti-S titre sub-groups “high” vs. “low.” A significant difference was not demonstrated between the anti-S titre sub-groups “negative” and “low” or “high” likely due to the small numbers of participants proving anti-S negative (*n* = 3), and thus, robust conclusions from this result are difficult to draw. The PRECISE group has previously demonstrated that ACE2-RBD binding inhibition is highest in anti-S plus anti-N positive individuals (28), and this is again demonstrated here via the statistical correlation between anti-N titres and ACE2-RBD binding inhibition when assessed via Spearman rank correlation or anti-N titre sub-group assessment via a Kruskal–Wallis test with Dunn's multiple comparison test. Multiple other factors influence antibody neutralisation capacity, including COVID-19 vaccination/infection history (28) and other SARS-CoV-2 neutralising antibody targets, such as the N-terminal domain (NTD) (6). It appears that this LFA POC cannot be relied upon solely to determine the neutralisation capacity of detected antibodies for a given individual. The decision to administer a COVID-19 vaccine dose may be influenced in a similar manner to COVID-19 therapeutics (above) by an individual failing to demonstrate any anti-S immune response to prior vaccination or infection as assessed via LFA, i.e., a “Negative” result. However, as the rationale for booster COVID-19 vaccine doses following primary vaccine schedules is based on post-dose increases in NAb levels from baseline (56) and as there was no significant difference in ACE2-RBD binding inhibition demonstrated by LFA result categories, conclusions cannot be drawn from these results in relation to booster COVID-19 vaccine doses in those with “Weak Positive” or “Positive” results via LFA.

## Limitations

There are a number of limitations that need to be considered in the current study. The Roche Elecsys-S assay determines total anti-Spike IgG/A/M in participant sera, while the Healgen Rapid Test Cassette LFA identifies anti-spike-RBD antibodies specifically. While correlation between the tests is demonstrated above, the authors acknowledge that the variation in target may contribute to the differences demonstrated in correlation with the pseudo-neutralisation assay. The upper limit of quantification for the Roche Elecsys-S antibody assay is 250 IU/mL, and further quantification of anti-S titres above this level was not logistically possible. A significant proportion of participants demonstrated titres exceeding this limit, and while a correlation between anti-S titres and ACE2-RBD inhibition is demonstrated, the exact titre level at which this occurs statistically was not determined. This limitation has been noted in other studies using commercial assays (57). The ACE2-RBD binding inhibition assay utilises a SARS-CoV-2 spike protein-RBD based on the wild-type Wuhan Hu-1 strain, and therefore, the authors cannot comment on the neutralisation capacity of measured antibodies against other variants of concern (VOC). Additionally, it is noted that this assay will not identify NAbs that target sites other than RBD; for example, those targeted towards the NTD.

## Conclusion

As the COVID-19 pandemic progresses from pandemic to endemic stage with a concurrent reduction in the extent of SARS-CoV-2 testing, LFA POC such as the Healgen Rapid Test Cassette are useful tools in sero-epidemiology settings and may act as supportive tools in aiding clinical treatment decisions through the rapid identification of anti-spike antibodies.

This POC LFA is not a reliable determinant of the neutralisation capacity of identified antibodies despite detecting antibodies known to have this function. Further data in relation to absolute antibody titres, patient SARS-CoV-2 infection/vaccination history, and performance of functional neutralisation assays are required to gain insight into this function and the resultant immune protection for a given individual.

## Data availability statement

The raw data supporting the conclusions of this article will be made available by the authors, without undue reservation.

## Ethics statement

The studies involving humans were approved by National Research Ethics Committee (NREC). The studies were conducted in accordance with the local legislation and institutional requirements. The participants provided their written informed consent to participate in this study.

## Author contributions

JM and LO'D aided in the study design, study execution, data analysis, and wrote the manuscript. CF and CB acted as site leads in each site and oversaw and contributed to the study design, execution, data analysis, and manuscript review. NC, JD, GB, AI, and WM undertook laboratory analysis, contributed to the study design, and reviewed the manuscript. SW contributed to the study execution, data analysis, and reviewed the manuscript. LD and CW undertook data analysis and reviewed the manuscript. NA and CK reviewed the manuscript. The PRECISE steering group aided in the study design and study execution. All authors reviewed the final manuscript.

## PRECISE Study (Prevalence of COVID-19 in Irish Healthcare Workers) Steering Group Members and Affiliations

LO'D, National Clinical Director for Health Protection, HSE Health Protection Surveillance Centre (HPSC), Dublin, Ireland, and Chair of Steering Group, NA, Consultant Physician in Infectious Diseases, JM, Consultant Physician in Infectious Diseases, CB, Consultant Physician in Infectious Diseases and Site Lead for PRECISE study, St. James's Hospital, Dublin, Ireland, NC, Consultant Immunologist, St. James's Hospital, Dublin, Ireland, LD, Surveillance Scientist, HSE-HPSC, Dublin, Ireland, CF, Consultant in Infectious Diseases and Site Lead for PRECISE study, Galway University Hospital, Galway, Ireland, Dr. Margaret Fitzgerald, National Public Health Lead, National Social Inclusion Office, Dublin, Ireland, Dr. Cillian de Gascun, Director, UCD National Virus Reference Laboratory, University College Dublin, Dublin, Ireland, Joan Gallagher, Programme Manager, Office of the National Clinical Director for Health Protection, HSE-HPSC, Dublin, Ireland, Dr. Derval Igoe, Specialist in Public Health Medicine, HSE HPSC, Dublin, Ireland, Prof. Mary Keogan, Consultant Immunologist Beaumont Hospital and Clinical Lead, National Clinical Programme for Pathology, HSE, Ireland, Dr. Noirin Noonan, Consultant in Occupation Medicine, St. James's Hospital, Dublin, Ireland, Professor Cliona O'Farrelly, Chair in Comparative Immunology, Trinity College Dublin, Ireland, and Dr. Breda Smyth, Department of Public Health, HSE West, Ireland.
